# Bm86 homologues and novel ATAQ proteins with multiple epidermal growth factor (EGF)-like domains from hard and soft ticks^☆^

**DOI:** 10.1016/j.ijpara.2010.06.003

**Published:** 2010-12

**Authors:** Ard M. Nijhof, Jesper A. Balk, Milagros Postigo, Anne Marie Rhebergen, Amar Taoufik, Frans Jongejan

**Affiliations:** a Utrecht Centre for Tick-Borne Diseases (UCTD), Faculty of Veterinary Medicine, Utrecht University, Yalelaan 1, 3584 CL Utrecht, The Netherlands; b Department of Tropical Diseases, Faculty of Veterinary Science, University of Pretoria, Private Bag X04, 0110 Onderstepoort, South Africa

**Keywords:** Bm86, Homologues, ATAQ, Tick, Vaccine, RNA interference, Expression profile

## Abstract

Tick control on livestock relies principally on the use of acaricides but the development of acaricide resistance and concerns for environmental pollution underscore the need for alternative control methods, for instance through the use of anti-tick vaccines. Two commercial vaccines based on the recombinant Bm86 protein from *Rhipicephalus* (*Boophilus*) *microplus* ticks were developed. Partial protection of the Bm86 vaccine against other *Rhipicephalus* (*Boophilus*) and *Hyalomma* tick species suggests that the efficacy of a Bm86-based vaccine may be enhanced when based on the orthologous recombinant Bm86 antigen. We therefore identified and analysed the Bm86 homologues from species representing the main argasid and ixodid tick genera, including two from the prostriate *Ixodes ricinus* tick species. A novel protein from metastriate ticks with multiple epidermal growth factor (EGF)-like domains which is structurally related to Bm86 was identified by using a 3′ rapid amplification of cDNA ends (3′-RACE) method with a degenerate primer based on a highly conserved region of Bm86 and its orthologues. This second protein was named ATAQ after a part of its signature peptide. Quantitative reverse transcriptase-PCR showed that ATAQ proteins are expressed in both midguts and Malpighian tubules, in contrast to Bm86 orthologues which are expressed exclusively in tick midguts. Furthermore, expression of this protein over the life stages of *R. microplus* and *Rhipicephalus appendiculatus* was more continuous compared with Bm86. Although a highly effective vaccine antigen, gene silencing of Bm86 by RNA interference (RNAi) produced only a weak phenotype. Similarly the RNAi phenotype of *Rhipicephalus evertsi evertsi* females in which the expression of *Ree86*, *ReeATAQ* or a combination of both genes was silenced by RNAi did not differ from a mock-injected control group. The vaccine potential of ATAQ proteins against tick infestations is yet to be evaluated.

## Graphical abstract

**Figure:**
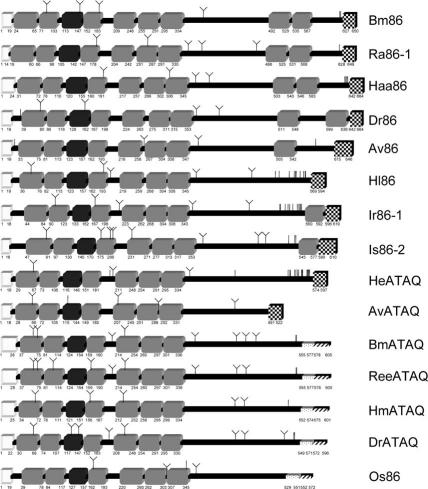

## 1 Introduction

Ticks are obligate hematophagous ectoparasites which can be divided into three families. The hard ticks or Ixodidae form the largest family which can be further subdivided into two groups, the basal Prostriata which consists of the genus *Ixodes*, and the more recent genera of the Metastriata. The soft ticks or Argasidae form a smaller family which is considered to be more basal than the Ixodidae. The third family, the Nuttalliellidae, is monotypic (Nava et al., 2009). Approximately 10% of all tick species have significant medical or veterinary importance by causing direct damage or production loss through blood feeding, by injecting toxins or by acting as vectors for a broad range of pathogens. The damage caused by ticks has considerable economic impact, in particular in the tropics and subtropics (Jongejan and Uilenberg, 2004). Control of ticks worldwide relies principally on the use of acaricides but concerns about environmental pollution, residues in food products and the development of acaricide resistance have resulted in the search for alternative means of tick control such as anti-tick vaccines. It has been known for over 70 years that immunity to ticks can be induced by vaccination with tick tissue homogenates and many studies have since focused on the identification and characterisation of tick-protective antigens, giving the vaccinated animal a certain degree of protection against tick infestations (Willadsen, 2004; de la Fuente et al., 2008).

In the 1990s, this led to the development and commercialisation of two related anti-tick vaccines targeting the common cattle tick *Rhipicephalus* (*Boophilus*) *microplus*: TickGARD Plus® in Australia and Gavac® in Cuba (de la Fuente et al., 2007). These were the first, and remain the only commercially available, anti-parasite vaccines using a recombinant antigen. Bm86, the recombinant antigen on which both vaccines are based, was identified through a complex series of protein fractionations followed by vaccination trials in cattle to assess the antigenic efficacy against *R. microplus* (Willadsen et al., 1988, 1989). Bm86 is a glycoprotein of unknown function which is located predominantly on the surface of tick midgut digest cells (Gough and Kemp, 1993). Vaccination with recombinant Bm86 typically leads to a maximum reduction of 50% in the number of *R. microplus* ticks engorging on vaccinated animals, lower engorgement weights and a decrease in the number of oviposited eggs. The impact of vaccination on reproductive performance is only seen in the second and subsequent tick generations by a reduced number of larvae in the field (Willadsen, 2004). Bm86-based vaccines give a high protection efficacy (>99% reduction on the number of engorging ticks) against *Rhipicephalus* (*Boophilus*) *annulatus* infestations (Fragoso et al., 1998; Pipano et al., 2003; Canales et al., 2009), partial cross-protection against several other tick species, e.g. *Rhipicephalus* (*Boophilus*) *decoloratus*, *Hyalomma anatolicum anatolicum* and *Hyalomma dromedarii*, but do not work against *Amblyomma cajennense*, *Amblyomma variegatum* and *Rhipicephalus appendiculatus* (de Vos et al., 2001; Odongo et al., 2007; Rodríguez and Jongejan, unpublished data). Vaccination with rHaa86, the recombinant Bm86 homologue protein from *Hy. a. anatolicum*, resulted in a significant decrease in the number of engorging *Hy. a. anatolicum* larvae and females (Azhahianambi et al., 2009). The inefficacy of Bm86 vaccines against some tick species and absence of a direct knock-down effect are the main disadvantages of these vaccines and justify the development of improved vaccine formulations, for instance by combining multiple tick-protective antigens. It is reasonable to assume that protection with the homologous form of Bm86 in each tick species will be better than heterologous cross-protection, despite the finding that the efficacy of vaccination against *R. annulatus* infestations with the recombinant homologue of *R. annulatus* (Ba86) was lower than that with Bm86 (Canales et al., 2009). *Bm86* homologues from *R. annulatus*, *R. decoloratus*, *R. appendiculatus*, *Rhipicephalus sanguineus* (GenBank Accession No. EF222203), *Hy. anatolicum* and *Haemaphysalis longicornis* have previously been sequenced (de Vos et al., 2001; Liao et al., 2007; Odongo et al., 2007; Nijhof et al., 2009). This study was designed to characterise the Bm86 homologues from a broader range of ixodid and argasid tick species of veterinary and medical importance and revealed a novel group of potential anti-tick vaccine candidates.

## 2 Materials and methods

### 2.1 Ticks and tick feeds

*Ornithodoros savignyi* adults originating from Upington, Northern Cape province, South Africa were provided by the Department of Biochemistry, University of Pretoria, South Africa. The *O. savignyi* colony was maintained by regular artificial feeding (Schwan et al., 1991). Tick strains of *Amblyomma variegatum* (the Gambia), *Dermacentor reticulatus* (Noord-Brabant, The Netherlands), *Dermacentor variabilis* (United States), *Haemaphysalis elliptica* (South Africa), *Hyalomma marginatum* (Ajaccio, Corsica), *Ixodes ricinus* (The Netherlands) and *Rhipicephalus evertsi evertsi* (Kwazulu Natal, South Africa) were maintained on rabbits and cattle in the tick rearing facility of the Utrecht Centre for Tick-borne Diseases (UCTD). All tick feeds were approved by the Animal Experiments Committee (DEC) of the Faculty of Veterinary Medicine, Utrecht University (DEC No. 2008.II.07.068).

### 2.2 Tick dissections and RNA isolation

While submerged in autoclaved ice-cold PBS, pH 7.4, partially fed ixodid females fed on calves were halved between leg pairs 2 and 3 using a sterile scalpel blade. Field-collected *O. savignyi* females were immobilized in paraffin wax, submerged in autoclaved ice-cold PBS and their integument was removed by an incision with a sterile scalpel blade around the lateral margin of the body. Separate tissues were subsequently collected from the body of ixodid and argasid ticks using watchmaker’s forceps under a stereo microscope, transferred to 1 ml TRIzol (Invitrogen, Breda, The Netherlands) and homogenised by passage through 24- and 27-gauge needles. For the isolation of total RNA from unfed first stage nymphs (N_1_) from *O. savignyi*, pools of 100 mg *O. savignyi* N_1_ were homogenised in 1 ml TRIzol reagent using a Potter–Elvejhem glass/Teflon homogeniser. All samples collected in TRIzol were centrifuged at 12,000*g* at 4 °C for 10 min to remove insoluble material after which the supernatant was frozen at −80 °C until RNA extraction. Total RNA was isolated and treated with DNase I (Fermentas GmbH, St. Leon Rot, Germany) prior to purification using the Nucleospin RNA II kit (Machery-Nagel, Düren, Germany), all in accordance with the manufacturer’s protocols. Sample concentrations and purity were determined with a NanoDrop ND-1000 spectrophotometer (NanoDrop Technologies, Wilmington, DE, USA) at 260 nm (A260) wavelength.

### 2.3 cDNA synthesis and rapid amplification of cDNA ends (3′- and 5′-RACE)

For the 3′-RACE of Bm86 homologues, 1 μg of total RNA was used to synthesise first-strand cDNA using SuperScript III (Invitrogen) following the manufacturer’s instructions using a 3′-RACE anchor primer containing a poly-T sequence [5′-GCTATCATTACCACAACACTCT_(18)_(AGC)(AGCT)-3′]. This and all other primers used in this study were synthesised by Invitrogen, Paisley, UK. The *Bm86* orthologues of *D. reticulatus* (*Dr86*), *Hy. marginatum* (*Hm86*) and *R. e. evertsi* (*Ree86*) were subsequently PCR amplified from this cDNA using GoTaq Hot Start consumables (Promega, Leiden, The Netherlands) with degenerate primer Ra86-F [5′-TCATC(CT)(AG)T(CT)TGCTCTGACTTCGG-3′] and a 3′-RACE anchor primer [5′-GCTATCATTACCACAACACTC-3′].

The same strategy was used for amplification of the *I. ricinus* Bm86 orthologues (*Ir86-1* and *Ir86-2*) using forward primers Is86-1F [5′-TCCCCTGTCCTTGGATTGG-3′] and Is86-2F [5′-CAGCCAAGACATACCATAACG-3′] the designs of which were based on expressed sequence tag (EST) sequence information for the *Ixodes scapularis* Bm86 homologues discovered by a BLAST search of the database made available at the *I. scapularis* vectorbase website (http://iscapularis.vectorbase.org/Tools/BLAST/) (*Is86-1*: alignment of EW846881, EW825613 and EW943081; *Is86-2*: alignment of EW929369, EW893350, and EW858856) of the *I. scapularis* genome project (Pagel Van Zee et al., 2007). The conserved Bm86 peptide sequence (RCCQGWN, pos. 173–179 of Bm86, GenBank Accession No. AAA30098) was used to design degenerate primer Bm86 catchall-F [5′-CGITG(CT)TG(CT)CA(AG)GG(AG)TGG(AG)AC-3′] which amplified the partial Bm86 orthologues from *A. variegatum* (*Av86*) and *O. savignyi* (*Os86*) when used in combination with the 3′-RACE anchor primer.

A second protein, referred to as ATAQ later in this manuscript, was also amplified from *A. variegatum* by the Bm86 catchall-F primer. Based on this sequence and additional ESTs from the *R. microplus* EST database (http://compbio.dfci.harvard.edu/tgi/tgipage.html), additional primer ATAQ catchall-F [5′-ACIGCTCA(GA)CGATGCTACCA-3′] was developed for 3′-RACE of the ATAQ homologues from *R. annulatus*, *R. decoloratus*, *R. microplus*, *R. e. evertsi*, *R. appendiculatus*, *Hy. marginatum*, *D. reticulatus*, *D. variabilis* and *Hae. elliptica*.

The resulting sequences from the 3′-RACE reactions were used to design 5′-RACE primers for 5′-RACE. The 5′-RACE cDNA synthesis was conducted with 1 μg total RNA of each species using a second generation RACE kit (Roche Applied Science, Almere, The Netherlands) in accordance with the manufacturer’s protocols, followed by two PCRs with the 5′-RACE anchor primer [5′-GACCACGCGTATCGATGTCGAC-3′] from this kit and the primers shown in Supplementary Table S1. The PCR products were purified using the Nucleospin Extract kit (Machery-Nagel, Düren, Germany), cloned into the pGem-T easy vector (Promega) and sequenced by Baseclear, Leiden, The Netherlands. All sequences have been submitted to GenBank and can be retrieved under the respective accession numbers shown in Table 1.

### 2.4 Gene sequence and phylogenetic analysis

Sequence alignments were created using the BioEdit sequence alignment editor program (http://www.mbio.ncsu.edu/BioEdit/bioedit.html) with ClustalW. An identity/similarity matrix was generated using MatGAT v2.01 (Campanella et al., 2003). Signal peptides were predicted by SignalP (http://www.cbs.dtu.dk/services/SignalP/) and *N*-glycosylation and *O*-glycosylation of the deduced protein sequences were predicted by the NetNGlyc 1.0 (http://www.cbs.dtu.dk/services/NetNGlyc/) and NetOGlyc 3.1 (http://www.cbs.dtu.dk/services/NetOGlyc/) servers of the Center for Biological Sequence Analysis (CBS), Technical University of Denmark. The predicted molecular weight and the p*I* were determined using the Compute pI/MW tool of the ExPASy proteomics server (http://www.expasy.ch/tools/pi_tool.html). Potential glycosyl-phosphatidylinositol (GPI) anchor sites and transmembrane (TM) helices were predicted using the predGPI GPI predictor tool (http://gpcr.biocomp.unibo.it/predgpi/) and the TMHMM server (http://www.cbs.dtu.dk/services/TMHMM/). Proteins were scanned for repeat regions using RADAR (http://www.ebi.ac.uk/Tools/Radar/index.html).The annotated Is86-1 and Is86-2 genes were blasted against the assembled *I. scapularis* supercontigs (version IscaW1) of the *I. scapularis* genome sequencing project on Vectorbase (www.vectorbase.org/Tools/BLAST). Phylogenetic trees were generated using Treecon (Van de Peer and De Wachter, 1993). Antigenic peptides were predicted using the method of Kolaskar and Tongaonkar (1990), with a reported accuracy of approximately 75% (http://imed.med.ucm.es/Tools/antigenic.pl).

### 2.5 Expression analysis by quantitative reverse transcriptase-PCR (qRT-PCR)

cDNA was synthesised from 500 ng of DNA-free RNA isolated from tissues of adult *A. variegatum*, *I. ricinus*, *R. microplus* and *O. savignyi* ticks using the iScript cDNA synthesis kit (Bio-Rad, Veenendaal, The Netherlands) according to the manufacturer’s directions and stored at −20 °C until use in qRT-PCR. qRT-PCR assays using SYBR® green detection were designed and optimised for the amplification of the reference genes elongation factor 1α (*ELF1A*) and TATA box binding protein (*TBP*) and members of the Bm86 protein family: *Av86*, *AvATAQ*, *Ir86-1*, *Ir86-2*, *BmATAQ* and *Os86*. The methodology of the Bm86 qRT-PCR assay was published previously (Nijhof et al., 2009). Real-time analysis was carried out on an iCycler thermal cycler (Bio-Rad). RT-PCR amplification mixtures (25 μl) contained cDNA generated from 5 ng of RNA template, 12.5 μl MAXIMA™ SYBR green qPCR mastermix (Fermentas) and 400 nM forward and reverse primer. The cycling conditions comprised a 5 min denaturation and polymerase activation step at 95 °C, 40 cycles of 95 °C for 10 s, 60 °C for 30 s and 72 °C for 30 s. Upon completion of the amplification program, a dissociation analysis (52–95 °C) was performed to determine the purity of the PCR amplicons. To estimate amplification efficiencies, a standard curve was generated for each primer pair based on known quantities of cDNA for the corresponding tick species (10-fold serial dilutions corresponding to cDNA transcribed from 50 to 0.05 ng of total RNA in triplicate) and analyzed using the iQ 5 software (Bio-Rad). The expression data were normalised using the geometric mean of the selected reference gene quantities and their respective amplification efficiencies (Nijhof et al., 2009). Normalised quantities were rescaled to the expression of the partially fed female midgut sample for comparison purposes and are shown as the mean ± SD in Figs. 3–5. All assays included this standard curve, a no-template control and each of the test cDNAs. Primers, amplicon lengths and PCR efficiencies are indicated in Supplementary Table S2.

### 2.6 RNA interference (RNAi)

Oligonucleotide primers RsATAQ double-stranded RNA (dsRNA) F1 (5′-TAATACGACTCACTATAGGCGAGAACTCATCAAATCCTTACTAC-3′), RsATAQ dsRNA R1 (5′-TAATACGACTCACTATAGGATTCTGTTCAATAGTGCTGGTGC-3′), Bm86h-F3T7 and Bm86h-R3T7 (Nijhof et al., 2007) all containing T7 promotor sequences at the 5′-end for in vitro transcription and synthesis of dsRNA were used to PCR-amplify cDNA from *R. e. evertsi* encoding *ReeATAQ* (548 bp) and *Ree86* (421 bp), respectively. PCR products were purified using the Nucleospin Extract kit (Machery-Nagel) and used as templates to produce dsRNA using the T7 Ribomax Express RNAi system (Promega, Leiden, The Netherlands). dsRNA aliquots were stored at −80 °C until used. For the injection of dsRNA, three groups of 20 *R. e. evertsi* females each were placed on double-sided sticky tape with the ventral sides upwards and injected into the base of the fourth leg on the right ventral side with 0.5 μl *Ree86*, *ReeATAQ* or a combination of *Ree86* and *ReeATAQ* dsRNA (5–7 × 10^11^ molecules/μl) using a 10 μl syringe with a 33 G needle (Hamilton, Bonaduz, Switzerland) mounted on a MM3301-M3 micromanipulator (World Precision Instruments (WPI), Berlin, Germany) and connected to an UMPII syringe pump (WPI). The tip of a 27 G needle was used to slightly pierce the integument before the 33 G needle was inserted. The dsRNA was dissolved in injection buffer (10 mM Tris–HCl, pH 7 and 1 mM EDTA). A fourth control group (*n *= 20) was injected with injection buffer alone. The ticks were placed in an incubator at 27 °C with 95% relative humidity for 4–6 h following injection, before they were examined for mortality and placed in four separate patches, one for each group, on calf #9918. Twenty-five male ticks were placed in each patch simultaneously with the injected females. The ticks were checked twice daily and collected when they dropped from the host. All ticks were weighed separately within 1 h of collection and stored individually in 15 ml jars with pierced lids at 27 °C and 95% relative humidity for oviposition. For gene expression analysis by qRT-PCR, total RNA was isolated from the guts of six partially fed females from each group collected at day 5 p.i. Biological triplicates were created for each group by dividing these six guts into three tubes, pooling two guts in each tube filled with TRIzol. RNA isolation, DNAse treatment and cDNA synthesis were performed as described in Sections 2.2 and 2.5. The primer combinations used for the qRT-PCR are shown in Supplementary Table S2.

### 2.7 Statistical analyses

Statistical analyses of data from the qRT-PCR and RNAi experiments were performed using Microsoft Excel as previously described (Nijhof et al., 2007, 2009). In short, gene expression levels were normalised using the geometric mean of selected reference gene quantities in Microsoft Excel following the guidelines described in the geNorm manual (http://medgen.ugent.be/~jvdesomp/genorm/geNorm_manual.pdf) and the 95% confidence interval was calculated. Differential gene expression was considered significant when the 95% confidence interval of the mean normalised expression levels did not overlap (equivalent to *P *< 0.05). Statistical analysis of data from the weights of ticks after feeding and oviposited egg mass was performed using Microsoft Excel and consisted of an unpaired *t*-test with unequal variances. Tick mortality was compared between the dsRNA- and mock-injected ticks by *χ*^2^-test. *P* values of 0.05 or less were considered statistically significant.

## 3 Results

### 3.1 Characterisation of the Bm86 homologues

**Fig. 1:**
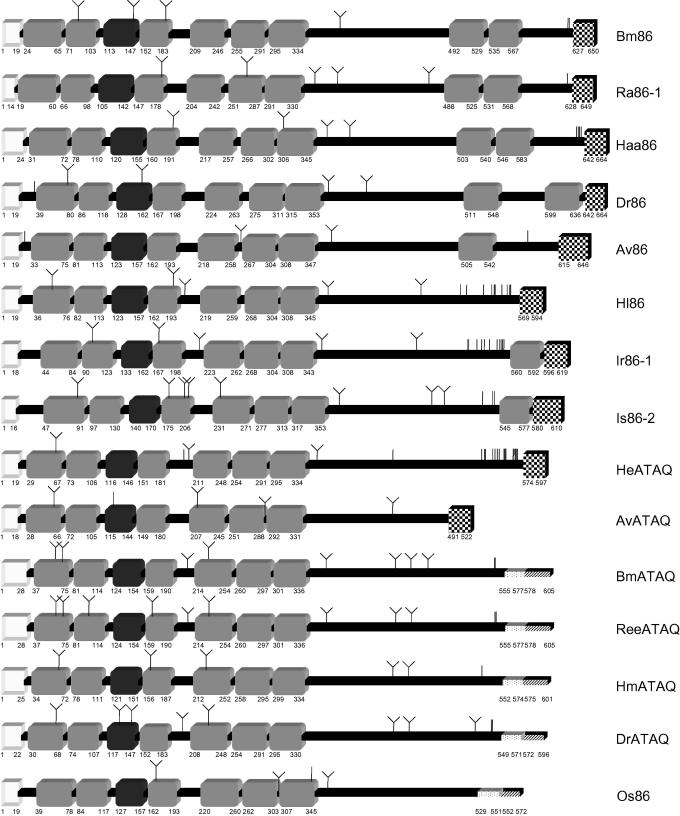
Comparison of the Bm86 and BmATAQ protein structure of *Rhipicephalus* (*Boophilus*) *microplus* (Australia) with representative Bm86- and ATAQ-orthologues from other tick genera: *Amblyomma variegatum* (Av86 and AvATAQ), *Dermacentor reticulatus* (Dr86 and DrATAQ), *Haemaphysalis elliptica* (HeATAQ), *Haemaphysalis longicornis* (Hl86), *Hyalomma anatolicum anatolicum* (Haa86), *Hyalomma marginatum* (HmATAQ), *Ixodes ricinus* (Ir8*6-1*), *Ixodes scapularis* (Is8*6-2*), *Ornithodoros savignyi* (Os86), *Rhipicephalus appendiculatus* (Ra8*6-1*) and *Rhipicephalus evertsi evertsi* (ReeATAQ). The signal peptides (white boxes), epidermal growth factor (EGF)-like domains (light grey boxes), partial EGF-like domains (dark grey boxes), glycosyl-phosphatidylinositol (GPI) anchors (checkered boxes), transmembrane (TM) domains (dotted highlights) and intracellular domains (striped highlights) are indicated. Potential *O*-linked carbohydrate additions are indicated by a vertical line, potential *N*-linked carbohydrate additions by a Y symbol. The numbers corresponds to the amino acid (AA) positions of the start and end of each protein domain.

The Bm86 homologues from the metastriate ticks *A. variegatum* (*Av86*), *D. reticulatus* (*Dr86*), *Hy. marginatum* (*Hm86*) and *R. e. evertsi* (*Ree86*) were successfully amplified using 3′- and 5′-RACE PCR and subsequently sequenced. The translated proteins showed structures similar to Bm86 with a signal peptide, multiple EGF-like domains fitting the pattern C-x(3, 9)-C-x(3, 6)-C-x(8, 11)-C-x(0, 1)-C-x(5, 15)-C (where x is any amino acid [AA] except cysteine), multiple glycosylation sites and a GPI anchor (Fig. 1). Their size, predicted molecular weight, p*I*, glycosylation sites, number of EGF-like domains and membrane anchors are shown in Table 1. A single Bm86-like protein (Os86) of 570 AA with a signal peptide, six full and one partial EGF-like domain and a TM anchor was found to be expressed in nymphs and adults of *O. savignyi*, the only soft tick used in this study.

A second sequence coding for a Bm86-like protein from *A. variegatum* was discovered following sequencing of products from a 3′-RACE PCR with the Bm86 catchall primer. When the full sequence encoding for this protein was obtained by 5′-RACE PCR, the translated complete protein sequence showed only 40% similarity to the Australian Bm86 isolate from *R. microplus*. We therefore had two sequences from *A. variegatum*: one with 62% similarity and a second with only 40% similarity to Bm86, while the two *A. variegatum* sequences again showed only 38% similarity to each other (Table 2). Despite this difference at sequence level, the cysteine-rich protein was predicted to have a structure similar to Bm86 with multiple EGF-like domains and a GPI anchor. This suggested that we were dealing with a distinct, although related, protein. A tBLASTn search with this protein sequence revealed the presence of a similar protein in the *R. microplus* EST database. 3′- and 5′-RACE PCR were employed to amplify the gene coding for this protein from *R. annulatus*, *R. decoloratus*, *R. microplus*, *R. e. evertsi*, *Hae. elliptica*, *Hy. marginatum*, *D. reticulatus* and *D. variabilis*. An alignment of all AA sequences from this protein group showed the presence of a signature peptide: YFNATAQRCYH which largely overlaps with the first EGF-like domain. Part of this signature peptide, ATAQ, was chosen as a name for proteins from this group to distinguish them from Bm86 orthologues. Besides this signature peptide, all proteins in this group contain a signal peptide, a large number of cysteine residues, multiple glycosylation sites and six full and one partial EGF-like domain. The predicted anchoring does however differ; the proteins from *A. variegatum* (AvATAQ) and *Hae. elliptica* (HeATAQ) were predicted to contain a GPI anchor, species belonging to the Hyalomminae and Rhipicephalinae subfamilies were predicted to contain a transmembrane anchor (Fig. 1 and Table 1). From the brown ear tick *R. appendiculatus*, two different homologues (*RaATAQ-1* and *RaATAQ-2*) were found. The RaATAQ-2 protein contains a 44 AA gap compared with RaATAQ-1 with which it shares 90% overall similarity. An in silico prediction of antigenic peptides for Bm86 and BmATAQ did not result in the identification of common predicted antigenic regions with similarities higher than 60%.

Two Bm86 homologues from the prostriate tick *I. ricinus* (*Ir86-1* and *Ir86-2*) were sequenced following 3′- and 5′-RACE PCR with primers based on two Bm86-like sequences from the *I. scapularis* EST database. Their AA sequences show 49% and 45% similarity to the Australian Bm86 sequence, respectively and 59% similarity between each other (Table 2). Both proteins have seven full and one partial EGF-like domain and are predicted to contain a GPI anchor. The signature peptide found in the ATAQ proteins is not present in either of the sequenced Bm86 homologues from *I. ricinus*.

**Fig. 2:**
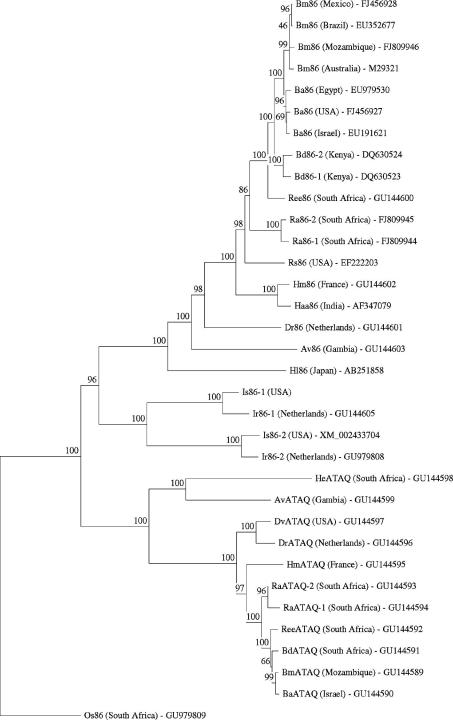
Neighbor-joining tree showing the phylogenetic relationship of the Bm86 protein family based on protein sequences without the signal peptides. The numbers represent the percentage of 1,000 replications (bootstrap support) for which the same branching patterns were obtained. The country of origin from each strain and GenBank Accession number are indicated. The Bm86 homologue from the soft tick *Ornithodoros savignyi* (Os86) was used as an outgroup.

Phylogenetic analysis separated both Ir86-1 and Ir86-2 from the Bm86 and ATAQ protein groups (Fig. 2). *Is86-1*, the homologue of *Ir86-1* from *I. scapularis*, was annotated from EST data and shares 92% identity on nucleotide level with *Ir86-1*. When a tBLASTn search against assembled *I. scapularis* supercontigs was performed to study the genomic organisation of *Is86-1* and *Is86-2*, *Is86-1* was shown to consist of 20 exons ranging from 48 to 171 bp in size and spanning >56 kb of genomic DNA. Exons 15 and 16 (AA 515–535 and 537–557) encode for repeats which are predicted to be extensively *O*-glycosylated. Similar repeats are also found in the *Ir86-1* (AA515–535 and 537–557), *Hl86* (BAF56919, AA 529–543 and 546–560) and *HeATAQ* (AA 531–549 and 552–570) sequences. The 19 exons of the annotated coding sequence of *Is86-2*, the *I. scapularis* homologue of *Ir86-2* with which it shares 93% identity on a nucleotide level, span >52 kb on the genome and range from 30 to 168 bp in size. The introns of Is8*6-1* and Is8*6-2* have a consensus GT/AG splice junction and average sizes of 3,346 bp (205–15,282 bp) and 3,119 bp (498–12,398 bp) respectively. Each of the first six full and one partial EGF-like domains of *Is86-1* and *Is86-2* is encoded by single exons whereas the last EGF-like domain is encoded by two exons (*Is86-1*: 17 and 18, *Is86-2*: 16 and 17). Sequence data for the region upstream of the start codon for Is8*6-1* is of poor quality in the IscaW1 assembly of supercontigs, but not for Is8*6-2* where a TATA box is found on position −59 to −54. The 3′ untranslated region (3′UTR) of both genes contain a polyadenylation signal (AATAAA).

BLAST analysis of all identified proteins belonging to the Bm86 family did not return significant hits other than the Bm86 homologues deposited in GenBank. The closest related proteins other than Bm86 are those with similar EGF or EGF-like domains such as latent transforming growth factor binding protein 4, fibrillin and matrilin.

### 3.2 Expression patterns of members of the Bm86 protein family

**Fig. 3:**
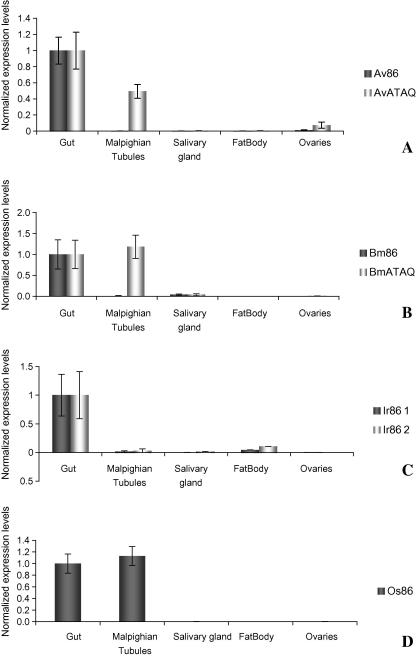
Quantitative reverse transcriptase-PCR analysis showing the transcription levels of *Av86* and *AvATAQ* (A), *Bm86* and *BmATAQ* (B), *Ir86-1* and *Ir86-2* (C) and *Os86* (D) in various tissues of *Amblyomma variegatum* (A), *Rhipicephalus* (*Boophilus*) *microplus* (B), *Ixodes ricinus* (C) and *Ornithodoros savignyi* (D). Bars represent the 95% confidence interval of the expression normalised reference genes elongation factor 1α (*ELF1A*) and TATA box binding protein (*TBP*).

Total RNA of various tissues from *A. variegatum*, *R. microplus*, *I. ricinus* and *O. savignyi* was screened by qRT-PCR with gene-specific primers for the expression of members of the Bm86 protein family. Expression levels were normalised using geometric averaging of reference genes ELF1A and TBP. The Bm86 homologues, Ir8*6-1* and Ir8*6-2*, were found to be transcribed almost exclusively in the midgut whereas ATAQ proteins and the Bm86-like protein from *O. savignyi* were expressed in both midgut and Malpighian tubules (Fig. 3).

**Fig. 4:**
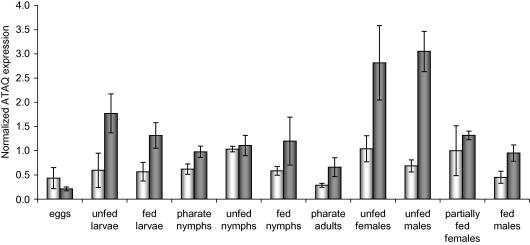
Relative BmATAQ (white bars) and RaATAQ (grey bars) protein expression levels in all life stages, normalised against the six most stably expressed reference genes in both *Rhipicephalus* (*Boophilus*) *microplus* and *Rhipicephalus appendiculatus*: elongation factor 1α (*ELF1A*), TATA box binding protein (*TBP*), glyceraldehyde 3-phosphate dehydrogenase (*GAPDH*), H3 histone family 3A (*H3F3A*), cyclophilin (*PPIA*), and ribosomal protein L4 (*RPL4*) (Nijhof et al., 2009). Bars represent the 95% confidence interval of the normalised expression.

The expression of BmATAQ and RaATAQ was also measured throughout all life stages of *R. microplus* and *R. appendiculatus*, respectively, by qRT-PCR and normalisation with multiple reference genes *ELF1A*, glyceraldehyde 3-phosphate dehydrogenase (*GAPDH*), H3 histone family 3A (*H3F3A*), cyclophilin (*PPIA*), ribosomal protein L4 (*RPL4*) and *TBP* (Fig. 4). BmATAQ was shown to be expressed constantly throughout the life cycle of *R. microplus* with limited variation. The expression of RaATAQ decreased slightly with feeding and molting of the juvenile life stages of *R. appendiculatus*. The highest RaATAQ expression levels were found in unfed adults, where expression also decreased during feeding in both females and males.

### 3.3 Gene silencing of Ree86 and ReeATAQ in *R. e. evertsi* females by RNAi

**Fig. 5:**
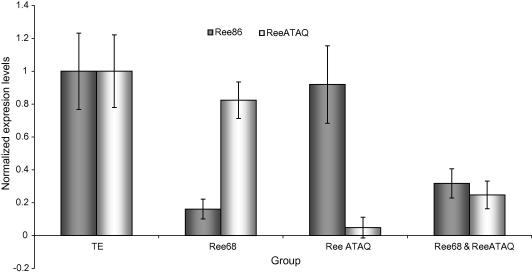
Quantitative real-time reverse transcriptase-PCR analysis showing the relative *Ree86* (grey bars) and *ReeATAQ* (white bars) transcript levels in the guts of partially fed females, 5 days after they were injected with injection buffer alone (TE), *Ree86*-, *ReeATAQ*-, or a combination of *Ree86*- and *ReeATAQ*-dsRNA, and fed on calf #9918. Bars represent the 95% confidence interval of the expression normalised against the elongation factor 1α (*ELF1A*), and TATA box binding protein (*TBP*) transcript levels.

A small scale RNAi experiment was performed to determine the effect of silencing the expression of Ree86 and ReeATAQ, both alone and in combination, on the feeding of *R. e. evertsi* females. Mortality, engorgement weight and oviposited egg mass did not differ significantly between the test and mock-injected control groups (Table 3). qRT-PCR on total RNA extracted from the guts of females from each group demonstrated that the target genes were successfully silenced (Fig. 5). *Ree86* expression levels normalised for the total amount of RNA used to generate the cDNA were 16% (± 29%) higher in the *ReeATAQ* dsRNA injected group compared with the control group. Similarly, the *ReeATAQ* expression levels were found to be 14% (± 5%) higher in the *Ree86* dsRNA injected group compared with the control group. These differences were not significant (*P *> 0.05) when the expression levels were normalised with reference genes *ELF1A* and *TBP*.

## 4 Discussion

In this study, the diversity of Bm86 homologues from representatives of the main tick genera was characterised using RACE strategies with primers based on available sequence information from GenBank and the *I. scapularis* genome project (Pagel Van Zee et al., 2007) followed by sequencing. The results of the phylogenetic analysis for these Bm86 orthologues (Fig. 2) are in general agreement with recent insights in the systematics of ticks with the Hyalomminae being embedded in the Rhipicephalinae (Nava et al., 2009) and a clear division between the homologues from metastriate and prostriate ticks.

Interestingly, the combination of bioinformatics and RACE strategies led to the discovery of novel proteins which are structurally related to Bm86 and may be potential anti-tick vaccine candidates based on this similarity: two Bm86 homologues occurring in the prostriate ticks *I. ricinus* (*Ir86-1* and *Ir86-2*) and *I. scapularis* (*Is86-1* and *Is86-2*) and the ATAQ protein group from metastriate ticks. ATAQ orthologues could not be identified in the partially assembled genome and EST database of *I. scapularis* or by 3′-RACE PCR using various degenerate primers on *I. ricinus* RNA (results not shown). The apparent lack of ATAQ orthologues in prostriate (*Ixodes*) ticks would indicate that these proteins developed after the divergence of Prostriata and Metastriata in acarine evolution. The combined results suggest that the evolution of the Bm86 protein family has been characterised by at least two gene duplication events: one in the prostriate lineage and a second in the metastriate lineage, resulting in the formation of the ATAQ protein group.

A clear function cannot be attributed to members of the Bm86 protein family due to a lack of significant similarity to proteins with known functions. All members of the Bm86 protein family have a large number of cysteine residues and contain several regions with similarity to EGF-like domains. The consensus sequence for these EGF-like domains from Bm86 was previously defined as C-x(4, 8)-C-x(3, 6)-C-x(8, 11)-C-x(0, 1)-C-x(5, 15)-C, based on the single sequence of Bm86 from *R. microplus* (Rand et al., 1989). The majority of the EGF-like domains found in members of the Bm86 protein family do fall within this definition. However, the first EGF-like domain from Ir8*6-2* and Is8*6-2* contains nine non-cysteine AA between its first two cysteine residues and the sixth EGF-like domain of BmATAQ contains only three non-cysteine AA between its first two cysteine residues. Based on these exceptions, the new consensus sequence for the EGF-like domains of the Bm86 family can be broadened to C-x(3, 9)-C-x(3, 6)-C-x(8, 11)-C-x(0, 1)-C-x(5, 15)-C.

Bm86 from *R. microplus* was demonstrated to be anchored to the cell membrane by a GPI anchor (Richardson et al., 1993). All Bm86 orthologues, the Bm86 homologues from the Prostriata and the ATAQ proteins of *A. variegatum* and *H. elliptica* are also predicted to be GPI anchored, whereas the ATAQ proteins of the Rhipicephalinae and Os86 are predicted to have a TM anchor (Fig. 1 and Table 1). Precedents exist in other protein families where some members are inserted into the cell membrane by a GPI anchor and others by a TM anchor, for example in the cadherin superfamily (Hulpiau and van Roy, 2009) and the carcinoembryonic antigen (CAE) gene family. In the CAE family, only a small number of mutations in the transmembrane domain resulted in a shift from transmembrane- to GPI-anchorage (Naghibalhossaini and Stanners, 2004). The GPI anchor in Bm86 orthologues may thus be derived from an ancestral transmembrane domain found in the ATAQ proteins of the Rhipicephalinae and Os86. The relevance of the GPI anchor of Bm86 orthologues and the ATAQ protein of *A. variegatum* and *H. elliptica* is unknown. Putative cellular functions of GPI anchors include involvement in (i) the partitioning of lipid rafts, subdomains of the cell membrane enriched in cholesterol, sphingolipid and GPI anchored proteins that organise the bioactivity of cell membranes (Lingwood and Simons, 2010), (ii) signal transduction, (iii) prion disease pathogenesis and (iv) acting as an apical-targeting signal (Paulick and Bertozzi, 2008). The latter function may provide an explanation for the demonstrated polarised distribution of Bm86 on the apical region of gut digest cells (Tellam et al., 1992). However, many GPI anchored membrane proteins function equally well when the GPI anchor is substituted by a TM proteinaceous anchor (Chatterjee and Mayor, 2001). Thus, it remains to be investigated if there is a functional difference between the GPI anchored and TM anchored ATAQ proteins.

The tissue distribution of the Bm86 protein family differs: ixodid Bm86 orthologues, including the homologues from *I. ricinus*, are expressed exclusively in the midgut but ATAQ proteins and argasid Bm86 homologue (Os86) can be found in both the midgut and MT (Fig. 3). Although it is currently not known which cells express ATAQ in the Malpighian tubules, cells expressing the related Bm86 protein during embryogenesis are thought to be stem cells and/or prodigest cells of the tick midgut (Nijhof et al., 2009). It is tempting to speculate that ATAQ may be expressed by stem cells of the tick midgut and Malpighian tubules in a similar fashion, associating it with cell growth or differentiation. Although multipotent stem cells have recently been identified in the Malpighian tubules of *Drosophila* spp. (Singh et al., 2007), it is not known whether this cell type is also present in the Malpighian tubules of ticks. Possible differences between prostriate and metastriate ticks that may explain the apparent lack of expression of a Bm86 protein family member in the Malpighian tubules of the prostriate *I. ricinus* (Fig. 3) have not been identified, as little is known about physiological processes occurring in the Malpighian tubules of ticks.

The expression of ATAQ proteins in both midgut and Malpighian tubules is of interest in the development of vaccines for the control of tick infestations. Intuitively, its structural similarity to Bm86, the midgut antigen on which commercial tick vaccines targeting *R. microplus* are based, suggests that vaccination with a recombinant ATAQ protein may confer protection against homologous tick infestations to a similar extent as vaccination with Bm86 by damaging the midgut. If so, this may result in an increased cross-protection against heterologous Rhipicephalinae tick infestations compared with that found for Bm86-based vaccines since the ATAQ proteins of the Rhipicephalinae are more conserved than this group’s Bm86 orthologues (Table 2). The in silico prediction of antigenic peptides for both Bm86 and BmATAQ, which share 44% overall similarity, did not result in the identification of common epitopes with significant similarity. Furthermore, regions of Bm86 previously identified as immunogenic (Patarroyo et al., 2002; Odongo et al., 2007) have no significant similarity with BmATAQ either (ranging from 9% to 29%), making it less likely that anti-Bm86 antibodies target BmATAQ as well. The expression of ATAQ in the Malpighian tubules could transform this organ into a potential second immunological attack site. Supporting data for the potential of the Malpighian tubules as a target tissue for an anti-tick vaccine comes from a recent vaccination trial in sheep targeting 5′-nucleotidase, an enzyme which is principally located in the Malpighian tubules. Vaccination with recombinant 5′-nucleotidase resulted in an overall egg mass reduction by a standard number of infesting *R. microplus* adults of 73% (Hope et al., 2010).

Although a highly effective vaccine antigen, gene silencing of Bm86 by RNAi in *R. microplus* did not result in a phenotype which was significantly different from that of the control group and gene silencing of its orthologue *Hl86* in *H. longicornis* produced only a weak phenotype (Liao et al., 2007; Nijhof et al., 2007). Similarly, the RNAi phenotype of *R. e. evertsi* females in which the expression of *Ree86*, *ReeATAQ* or a combination of both genes was silenced by RNAi did not differ from a mock-injected control group (Table 3). It has previously been hypothesised that the expression of functional paralogues of a silenced gene in the salivary glands of ticks may be induced to compensate for the loss of function caused by the RNAi, thus representing a “fall-back” strategy of the tick (Narasimhan et al., 2004). We could not observe a similar effect for Ree86 and ReeATAQ, i.e. no up-regulation of *Ree86* was found when *ReeATAQ* was silenced and vice versa. RNAi as conducted here thus failed to suggest a function for these proteins or to provide indirect evidence that Bm86 orthologues (*Ree86*) and ATAQ proteins (*ReeATAQ*) are functional paralogues, despite their structural similarities.

In conclusion, Bm86 homologues from various hard and soft tick species of veterinary and medical importance were isolated and sequenced. All Bm86 orthologues from hard ticks contain a signal peptide, six to eight EGF-like domains and a GPI anchor and are expressed in the midguts of ticks. A group of structurally similar proteins with a signal peptide, multiple EGF-like domains and a GPI- or TM anchor were identified in several metastriate tick species and named ATAQ after a part of their signature peptide. ATAQ proteins were found to be expressed in both the midgut and Malpighian tubules of ticks. The potential of these Bm86 orthologues and the ATAQ proteins as anti-tick vaccine candidates, alone or in combination, remains to be investigated.

## Figures and Tables

**Table 1:** Novel *Bm86* homologues and *ATAQ* genes identified in this study.

Gene	Tick species	GenBank Accession No.	AA No.	Molecular weight	p*I*	Glycosylation (*N*-linked/*O*-linked)	EGF domains (full/partial)	Anchor
*BmATAQ*	*Rhipicephalus* (*Boophilus*) *microplus*	GU144589	605	66.6	4.82	8/2	6/1	TM
*BdATAQ*	*Rhipicephalus* (*Boophilus*) *decoloratus*	GU144591	605	66.5	5.16	8/4	6/1	TM
*BaATAQ*	*Rhipicephalus* (*Boophilus*) *annulatus*	GU144590	605	66.4	5.05	8/2	6/1	TM
*ReeATAQ*	*Rhipicephalus evertsi evertsi*	GU144592	605	66.4	4.95	8/3	6/1	TM
*RaATAQ-1*	*Rhipicephalus appendiculatus*	GU144594	605	66.7	5.42	7/3	6/1	TM
*RaATAQ-2*	*R. appendiculatus*	GU144593	561	61.6	5.33	8/1	6/1	TM
*HmATAQ*	*Hyalomma marginatum marginatum*	GU144595	601	65.5	5.18	5/1	6/1	TM
*DrATAQ*	*Dermacentor reticulatus*	GU144596	596	64.7	4.79	9/2	6/1	TM
*DvATAQ*	*Dermacentor variabilis*	GU144597	598	65.0	4.84	6/4	6/1	TM
*HeATAQ*	*Haemaphysalis elliptica*	GU144598	597	65.6	5.47	3/17	6/1	GPI
*AvATAQ*	*Amblyomma variegatum*	GU144599	522	57.5	5.04	4/1	6/1	GPI
*Ree86*	*R. e. evertsi*	GU144600	680	75.1	6.29	4/0	8/1	GPI
*Dr86*	*D. reticulates*	GU144601	664	73.3	5.96	4/1	8/1	GPI
*Hm86*	*Hy. m .marginatum*	GU144602	664	72.3	6.23	4/6	8/1	GPI
*Av86*	*A. variegatum*	GU144603	650	72.1	5.66	2/2	7/1	GPI
*Ir86-1*	*Ixodes ricinus*	GU144605	619	68.0	6.33	5/10	7/1	GPI
*Is86-1*	*Ixodes scapularis*	Alignment of EW846881, EW825613, and EW943081	619	68.1	6.50	7/11	7/1	GPI
*Ir86-2*	*I. ricinus*	GU979808	610	68.4	7.22	7/7	7/1	GPI
*Is86-2*	*I. scapularis*	Alignment of EW929369, EW893350, and EW858856	610	68.6	6.95	8/3	7/1	GPI
*Os86*	*Ornithodoros savignyi*	GU979809	572	64.7	5.23	3/1	6/1	TM

AA, amino acid; EGF, epidermal growth factor; TM, transmembrane anchor; GPI, glycosyl-phosphatidylinositol anchor.

**Table 2:** Identity/similarity matrix of the Bm86 protein family. The numbers represent the percentage identity (in italics, upper right triangle) and similarity (lower left triangle) found between the full amino acid (AA) sequences.

	1	2	3	4	5	6	7	8	9	10	11	12	13	14	15	16	17	18	19	20	21	22	23	24
1. Bm86		*94*	*86*	*84*	*73*	*71*	*67*	*66*	*53*	*46*	*39*	*30*	*28*	*26*	*25*	*26*	*26*	*26*	*24*	*25*	*25*	*26*	*25*	*22*
2. Ba86	96		*88*	*86*	*74*	*72*	*67*	*66*	*53*	*47*	*40*	*30*	*29*	*27*	*26*	*26*	*26*	*26*	*25*	*25*	*25*	*26*	*24*	*22*
3. Bd8*6-1*	92	93		*87*	*75*	*72*	*68*	*67*	*53*	*48*	*40*	*30*	*28*	*26*	*24*	*25*	*25*	*25*	*24*	*24*	*24*	*25*	*23*	*22*
4. Ree86	89	91	93		*76*	*75*	*68*	*68*	*54*	*46*	*41*	*31*	*28*	*27*	*25*	*25*	*25*	*25*	*24*	*25*	*25*	*26*	*24*	*23*
5. Ra8*6-1*	81	82	84	85		*74*	*66*	*65*	*54*	*47*	*40*	*31*	*30*	*27*	*25*	*26*	*26*	*25*	*25*	*25*	*25*	*26*	*24*	*23*
6. Rs86	83	83	84	86	85		*68*	*69*	*56*	*49*	*42*	*30*	*30*	*28*	*25*	*25*	*25*	*25*	*24*	*24*	*25*	*26*	*23*	*22*
7. Hm86	78	78	79	80	78	81		*91*	*54*	*50*	*41*	*32*	*30*	*26*	*26*	*26*	*26*	*26*	*25*	*25*	*25*	*25*	*25*	*24*
8. Haa86	79	79	81	81	78	81	95		*52*	*48*	*42*	*31*	*30*	*27*	*27*	*26*	*26*	*25*	*26*	*26*	*26*	*25*	*24*	*24*
9. Dr86	68	68	68	70	68	72	69	69		*48*	*41*	*32*	*32*	*26*	*25*	*25*	*26*	*26*	*25*	*26*	*25*	*27*	*24*	*25*
10. Av86	62	63	63	63	63	65	63	63	66		*42*	*31*	*29*	*26*	*23*	*23*	*24*	*24*	*25*	*25*	*24*	*25*	*23*	*24*
11. Hl86	55	55	55	57	57	57	57	56	56	58		*33*	*30*	*30*	*29*	*29*	*29*	*29*	*29*	*29*	*28*	*29*	*26*	*25*
12. Ir8*6-1*	49	48	45	48	47	46	47	48	49	48	51		*44*	*33*	*26*	*26*	*26*	*26*	*28*	*28*	*27*	*28*	*28*	*27*
13. Ir8*6-2*	45	45	44	45	46	47	45	45	46	46	49	62		*30*	*28*	*27*	*28*	*27*	*27*	*28*	*28*	*28*	*25*	*26*
14. Os86	41	41	41	43	43	41	42	42	41	44	47	51	47		*29*	*28*	*30*	*29*	*29*	*30*	*30*	*29*	*28*	*28*
15. BmATAQ	44	45	42	43	43	44	44	45	43	43	48	44	44	46		*98*	*95*	*94*	*88*	*77*	*76*	*73*	*37*	*36*
16. BaATAQ	43	44	42	42	43	44	44	44	42	43	47	43	43	46	99		*95*	*94*	*87*	*77*	*75*	*72*	*37*	*36*
17. BdATAQ	44	43	41	42	43	43	43	44	42	43	47	43	43	46	98	98		*95*	*89*	*77*	*76*	*73*	*37*	*36*
18. ReeATAQ	43	44	42	43	43	44	44	44	42	44	47	43	43	46	98	97	98		*90*	*77*	*76*	*73*	*37*	*36*
19. RaATAQ-1	43	43	42	43	43	44	43	44	41	45	48	43	44	47	94	93	94	94		*79*	*75*	*73*	*38*	*36*
20. HmATAQ	43	44	41	43	43	42	43	42	43	43	47	45	44	47	88	87	87	88	88		*76*	*73*	*36*	*38*
21. DrATAQ	43	42	42	43	43	44	43	43	41	42	47	46	44	47	85	85	85	85	86	87		*89*	*37*	*36*
22. DvATAQ	42	42	41	41	42	43	42	42	43	43	47	45	44	46	84	84	84	84	85	85	94		*37*	*36*
23. AvATAQ	40	39	38	40	37	38	38	40	38	38	43	41	40	44	51	51	50	52	51	51	52	51		*40*
24. HeATAQ	40	38	40	40	39	39	41	41	42	42	44	45	41	46	54	54	53	54	52	54	54	54	53	

**Table 3:** Tick weight, mortality after feeding and egg mass weight in double-stranded RNA (dsRNA)-injected *Rhipicephalus evertsi evertsi* females. Each group consisted of 14 injected females.

Group	Tick weight (mg)	Mortality (%)	Eggs/tick (mg)
Control	684 ± 117	0	310 ± 64
*Ree86*	575 ± 130	0	229 ± 129
*ReeATAQ*	629 ± 123	0	275 ± 56
*Ree86* and *ReeATAQ*	671 ± 108	0	277 ± 92

## Appendix A Supplementary data

Supplementary Table S1 List of primers used for rapid amplification of cDNA ends (5′-RACE and 3′-RACE) in this study.

Supplementary Table S2 List of primers used for the quantitative real-time reverse transcriptase-PCRs in this study.
