# Supplementary material for: Bm86 homologues and novel ATAQ proteins with multiple epidermal growth factor (EGF)-like domains from hard and soft ticks
Source: Int J Parasitol. 2010 Dec;40(14):1587–97. doi: 10.1016/j.ijpara.2010.06.003 (PMC2998001; doi:10.1016/j.ijpara.2010.06.003)
Supplement: Supplementary Table S1 — List of primers used for rapid amplification of cDNA ends (5′-RACE and 3′-RACE) in this study. [file mmc1.doc]

Supplementary Table S1. List of primers used for rapid amplification of cDNA ends (5’-RACE and 3’-RACE) in this study.

| **Primer name** | **Sequence (5’🡪3’)** | **Location** | **Purpose** |
| --- | --- | --- | --- |
| Av86 5’-RACE R1 | CACTGACAGACGGCAGTCTTATTTC | 854-878 | 5’-RACE cDNA synthesis *Av86* |
| Av86 5’-RACE R2 | TACCAGGTGAGCAGTAAGTCCCATCT | 594-619 | 5’-RACE *Av86* - 1^st^ PCR |
| Av86 5’-RACE R3 | GCCTTTGAACAGTCGTTTTGGTCC | 564-587 | 5’-RACE *Av86* - 2^nd^ PCR |
| Av86 3’-RACE F1 | GAGATGCGTCATTTCATGGTGTTTG | -2-22 | 3’-RACE *Av86* complete fragment |
| AvATAQ 5’-RACE R1 | CCCTCTTCAAACACACATTCCTTTCCCA | 785-812 | 5’-RACE cDNA synthesis *AvATAQ* |
| AvATAQ 5’-RACE R2 | GCAGGGTTTTTTTTCTTCATCATTGC | 752-777 | 5’-RACE *AvATAQ* – 1^st^ PCR |
| AvATAQ 5’-RACE R3 | TGAACTCAGGTGACTTCTTATCAGTG | 660-685 | 5’-RACE *AvATAQ* – 2^nd^ PCR |
| AvATAQ 3’-RACE F1 | GCTTCCTCAGTGCCGTTTCATCC | 32-54 | 3’-RACE *AvATAQ* complete fragment |
| BDRATAQ 5’-RACE R1 | TTACATTCAACTCTAGC(CT)TGGCACG | 758-782 (B&R)  740-764 (D) | 5’-RACE cDNA synthesis B*mATAQ*, *DrATAQ*, *DvATAQ*, *RaATAQ* and *ReeATAQ* |
| BDRATAQ 5’-RACE R2 | CAGCGGTTATCCTCTCTCAG(CT)TT | 520-542 (B&R)  499-521 (D) | 5’-RACE *BmATAQ*, *DrATAQ*, *DvATAQ*, *RaATAQ* and *ReeATAQ* - 1^st^ PCR |
| BDRATAQ 5’-RACE R3 | GTCATGCCATGTATTCCAGAACAGTC | 307-332 (B&R)  286-311(D) | 5’-RACE *BmATAQ*, *DrATAQ*, *DvATAQ* and *RaATAQ* - 2^nd^ PCR |
| BmBaRa ATAQ-F | ATG(GA)GAA(GAT)AATGAACAACGAACG | 1-23 | 3’-RACE *BaATAQ, BdATAQ, BmATAQ*, *HmATAQ*, *RaATAQ* and *ReeATAQ* complete fragment |
| DrATAQ 3’-RACE F1 | GCCGATGAAACTCCCGATGATA | 64-85 | 3’-RACE *DrATAQ* and *DvATAQ* complete fragment |
| Dr86 5’-RACE R1 | TTTTCGTA(GA)ACGCATATTTGTCCC | 1566-1589 | 5’-RACE cDNA synthesis *Dr86* |
| Dr86 5’-RACE R2 | TTCCATCCCTGACAGCAACG | 562-581 | 5’-RACE *Dr86* – 1^st^ PCR |
| DrRee86 5’RACE R1 | CCCGAAGTCAGAGCA(AG)ACAG | 110-129 (Dr86), 140-159 (Ree86) | 5’-RACE *Dr86* and *Ree86* - 2^nd^ PCR |
| HeATAQ 5’-RACE R1 | GTTCAGGAAGATTTTGTCGTCAGGA | 840-864 | 5’-RACE cDNA synthesis *HeATAQ* |
| HeATAQ 5’-RACE R2 | ATGGTTTTGTCTCTACACCTGAATACG | 732-758 | 5’-RACE *HeATAQ* - 1^st^ PCR |
| HeATAQ 5’-RACE R3 | CGTCGTAGAGTTCTTTCCCTTCCG | 710-733 | 5’-RACE *HeATAQ* - 2^nd^ PCR |
| HeATAQ 3’-RACE F1 | CACTTGTCAGCGTATTCATCCTTGT | 14-38 | 3’-RACE *HeATAQ* complete fragment |
| Hm86 5’-RACE R1 | CGGCAACTTCGGATACAGCAT | 1282-1302 | 5’-RACE cDNA synthesis *Hm86* |
| Hm86 5’-RACE R2 | CGCACTTTCCGTCCAGTAGTTGTTGAT | 881-907 | 5’-RACE *Hm86* - 1^st^ PCR |
| Hm86 5’-RACE R3 | TTCCCCATTCACCGCAATCGCAC | 414-436 | 5’-RACE *Hm86* - 2^nd^ PCR |
| Ir86-2 5’-RACE R1 | TTGGTCATTGGTCGTTGGGGTA | 732-753 | 5’-RACE cDNA synthesis *Ir86-2* |
| Ir86-2 5’-RACE R2 | GTACATATCCAGTGGGGCAGAACG | 641-664 | 5’-RACE *Ir86-2* - 1^st^ PCR |
| Ir86-2 5’-RACE R3 | CTCGCAGCAACGGTCGTCCTT | 577-597 | 5’-RACE *Ir86-2* – 2^nd^ PCR |
| Ir86-2 3’-RACE F1 | ATGCGGTCGCTATGTTTGTTTG | 1-22 | 3’-RACE *Ir86-2* complete fragment |
| Ir86-1 5’-RACE R1 | GTTCCATCCTTGACAGCAGCGGT | 560-582 | 5’-RACE cDNA synthesis *Ir86-1* |
| Ir86-1 5’-RACE R2 | GCTTCTTGGCGGGTCCACAGTCG | 447-469 | 5’-RACE *Ir86-1* - 1^st^ PCR |
| Ir86-1 5’-RACE R3 | GTAACGGACCGCAAGACTGCCAATG | 256-280 | 5’-RACE *Ir86-1* – 2^nd^ PCR |
| Os86 5’-RACE R1 | GTTGCCAGAGCATTGTCCATTTCTTTCC | 885-912 | 5’-RACE cDNA synthesis *Os86* |
| Os86 5’-RACE R2 | GCACTTAGCCTTCTCCTCCGGGCTGCA | 736-762 | 5’-RACE *Os86* - 1^st^ PCR |
| Os86 5’-RACE R3 | TGTTCCCATCCTTGACAGCACC | 542-563 | 5’-RACE *Os86* - 2^nd^ PCR |
| Os86 3’-RACE F1 | AGCGGGGACCGTTTCGGATGAACAG | 51-74 | 3’-RACE *Os86* complete fragment |
| Ra86 5’-RACE R1 | CGACCTTGACGCATTTGTT | 1432-1450 | 5’-RACE cDNA synthesis *Ra86* and *Ree86* |
| Ra86 5’-RACE R2 | GCACCGTGTAGTAATACTCATTCAG | 1081-1104 | 5’-RACE *Ra86* and *Ree86* – 1^st^ PCR |
| Ra86 5’-RACE R3 | AGGAGCGGCTGAACAGTTTG | 563-582 | 5’-RACE *Ra86* – 2^nd^ PCR |
