# Supplementary material for: Bm86 homologues and novel ATAQ proteins with multiple epidermal growth factor (EGF)-like domains from hard and soft ticks
Source: Int J Parasitol. 2010 Dec;40(14):1587–97. doi: 10.1016/j.ijpara.2010.06.003 (PMC2998001; doi:10.1016/j.ijpara.2010.06.003)
Supplement: Supplementary Table S2 — List of primers used for the quantitative real-time reverse transcriptase-PCRs in this study. [file mmc2.doc]

Supplementary Table S2. List of primers used for the quantitative real-time reverse transcriptase-PCRs in this study.

| **Gene Name** | **GenBank Accession number** | **Forward primer** | **Reverse primer** | **Amplicon length (bp)** | **Efficiency (%)** |
| --- | --- | --- | --- | --- | --- |
| *BaATAQ, BmATAQ* & *RaATAQ* | **GU144589**, **GU144593** and **GU144594** | GCCAAGAATGCG(TC)CTACAAAG | GACATTTGAACGAGCACTCCTC | 101 | 105 |
| *Av86* | **GU144603** | ACGGATGACTTCAAGACAAGACTG | TTTCTGTCGCGGAACCCTTTT | 144 | 89 |
| *AvATAQ* | **GU144599** | CAATACAGTAAGCAGGACCGC | GTTCCTCTCCGCACTCAATCA | 129 | 90 |
| *Ir86-1* | **GU144605** | ACCGCTGCTGTCAAGGATGGA | ATCTGCGACATTTGCCGTGC | 127 | 92 |
| *Ir86-2* | **GU979808** | TGACAGGGTGCCTAAACTACAG | GCAGCAACGGTCGTCCTT | 79 | 100 |
| *Os86* | **GU979809** | GGACCAAGAACTGGCACATCAT | TCCTTCTTCCAACACACACTCTT | 108 | 76 |
| *Ree86* | **GU144600** | CGTCCCGACTTGACCTGC | AGGAGCGGCTGAACAGTTTG | 101 | 96 |
| *ELF1A* | **EW679365**, **CD797149**, **AF240836**, **EU574868** and **XM_002411102** | CGCAAGTCTGGCAAGTCTGA | AT(GA)CCACCAATCTTGTAGACG | 124 | 90 |
| *TBP* | **XM_002402081**, **CD780134** and **CV453818** | GCCAAGAGTGAAGAGCAGTC | AGGAACTTGGCGTCAAA(GA)C | 82 | 102 |
